# Elite male table tennis matches diagnosis using SHAP and a hybrid LSTM–BPNN algorithm

**DOI:** 10.1038/s41598-023-37746-1

**Published:** 2023-07-17

**Authors:** Honglin Song, Yutao Li, Xiaofeng Zou, Ping Hu, Tianbiao Liu

**Affiliations:** 1 College of Physical Education and Sports, Beijing Normal University, Beijing, 100084 China; 2 School of Physical Education, Jilin University, Jilin, 130015 China; 3 Microsoft, Beijing, 100080 China

**Keywords:** Engineering, Data mining, Machine learning

## Abstract

This study adopts a new approach, SHapley Additive exPlanation (SHAP), to diagnose the table tennis matches based on a hybrid algorithm, namely Long Short-Term Memory–Back Propagation Neural Network (LSTM–BPNN). 100 male singles competitions (8535 rallies) from 2019 to 2022 are analyzed by a hybrid technical–tactical analysis theory, which hybridizes the double three-phase and four-phase evaluation theories. A k-means cluster analysis is conducted to classify 59 players’ winning rates into three levels (high, medium, and low). The results show that LSTM–BPNN has excellent performance (MSE = 0.000355, MAE = 0.014237, RMSE = 0.018853, and ${\mathrm{R}}^{2}$ = 0.988311) compared with six typical artificial intelligence algorithms. Using LSTM–BPNN to calculate the SHAP value of each feature, the global results find that the receive-attack and serve-attack phases of the ending match have essential impacts on the mutual winning probabilities. Finally, case applications show that the SHAP can directly obtain each feature importance on one or more matches, which is more objective and reliable than the traditional simulation method. This research explores an innovative way to understand and analyze matches, and these results have implications for the performance analysis of table tennis and related racket sports.

## Introduction

In racket sports, players’ technical–tactical performance is a crucial factor in winning the game, and much research on singles matches of table tennis has focused on it for the past few decades^1–10^. Techniques in table tennis include the non-constraining techniques of serving and the constraining techniques of attacking, controlling and defending, the most representative of which are fast attacking, pushing and chopping^11^. Tactics combine different sports techniques which players use to achieve superiority in the matches. The technical–tactical performance in table tennis is complicated in matches. Researchers have categorized different strokes in each rally according to respective phases and performed notational analysis on each phase^3–5,12^. As a strong table tennis country, China has many excellent players, which mainly benefits from continuous analysis of relevant technical–tactical performances^13^. The most usual method or theory is the traditional three-phase evaluation theory^1^. Based on that, phase-based theories have been proposed to improve it, such as the four-phase evaluation theory^3^ and the double three-phase evaluation theory^7^ in the descriptive analysis range^14^.

With the rapid development of deep learning and machine learning, many models and algorithms have been applied to study table tennis matches^15–25^. Researchers have built models and adjusted input values through simulations to achieve technical–tactical diagnosis^15,16,18,19,21^ based on sports training and competition methods or theories. Data mining helps coaches organize more purposeful training programs for athletes during preparation and provides targeted coaching during competitions. It also uncovers critical aspects that are easily overlooked in the competition.

However, three problems are overlooked in previous studies about evaluating or diagnosing players’ technical–tactical performance. One of the drawbacks is that previous studies fail to explain how the interaction between two players impacts their performance in the match^10,26–28^. Current diagnosis models only account for one side’s performance in table tennis matches, which does not consider the mutual influence and interaction between the two sides. Meanwhile, there is a correlation between players’ technical–tactical performance and their ability levels and technical styles. Therefore, it may yield biased results by only diagnosing one side’s technical–tactical performance or ignoring both sides’ styles and abilities.

Another problem in the previous studies is that they have not considered the serial characteristics of the data in the selection of algorithms. Two improved theories are based on the traditional three-phase evaluation theory: the four-phase evaluation theory^3^ and the double three-phase evaluation theory^7^. The former reclassifies the match into four phases (i.e. serve-attack phase, receive-attack phase, stalemate I phase, and stalemate II phase). The latter adds the concept of match progression to the evaluation (i.e. the beginning, middle, and end phases), giving the serial character to the data collected and each phase. Meanwhile, the recurrent neural network (RNN) is proposed to solve contextual relations in serial data^29^, but it suffers from gradient explosion and lacks information preservation over long periods. In order to solve the above problem, a particular type of RNN, the Long Short-Term Memory (LSTM) neural network^30^ is proposed. Previous studies have adopted LSTM to deal with series data with excellent performance in finance, motion recognition, and wearables^31–33^. Therefore, it may be more appropriate to use LSTM to construct a diagnosis model for technical–tactical analysis data with serial characteristics, leading to higher accuracy for the model.

In addition, previous studies have focused too much on the performance of the models, but the importance of interpreting models has been neglected^34^. They have used simulation analysis to find the key influencing factors in matches based on artificial intelligence algorithms^17,21^, which significantly reduced the reliability of the diagnostic results. Fortunately, there have been many different approaches to increasing the interpretability of models. Among them, the SHapley Additive exPlanation (SHAP) is widely recognized, which uses local approximation and cooperative game theory to explore the interpretability of complex models^35^. Explaining the model through SHAP has been used in many industries with excellent results^36–39^. However, few studies have used the SHAP in sports performance analysis. SHAP can dramatically improve technical–tactical diagnosis applicability in table tennis.

Inspired by previous studies, this study adopts a hybrid algorithm, namely LSTM–BPNN to diagnose table tennis matches using SHAP method. In order to further embody the interaction between two players, this study hybridize the four-phase evaluation theory and the double three-phase evaluation theory to build an interactive technical–tactical model including two side players. Meanwhile, LSTM–BPNN is the most appropriate here because it has excellent performance and robustness in prediction compared with six typical artificial intelligence algorithms. In terms of SHAP, this study visualizes all features to understand the technical–tactical model and uses local interpretation to diagnose and analyze table tennis matches. Meanwhile, this study conducts the case application to compare with the simulation and SHAP analyses.

## Method

**Figure 1:**
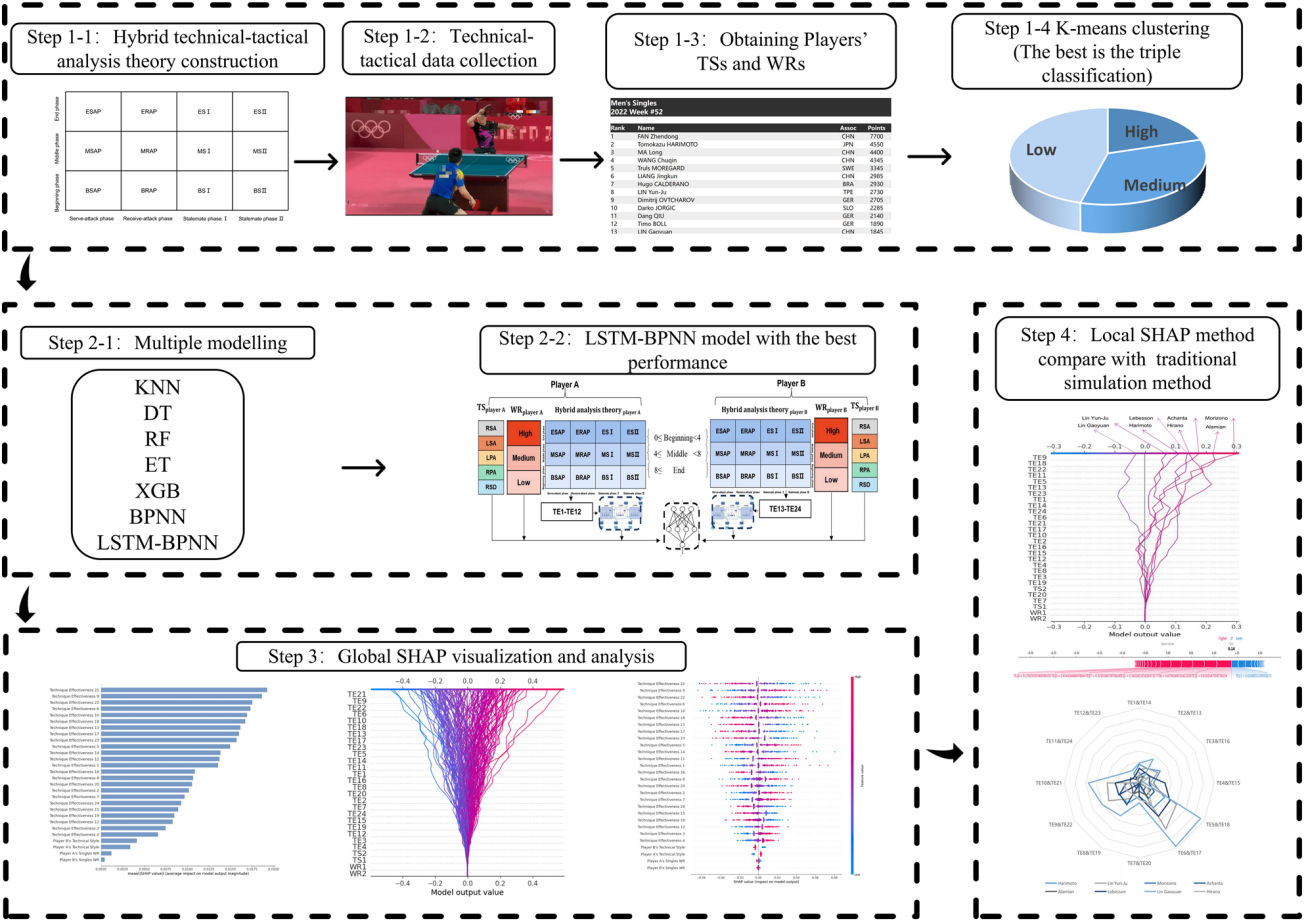
Study design. Step 1: data resources and preprocessing. Step 2: Multiple modelling and comparison. Step 3: Global SHAP visualization and analysis. Step 4: Local SHAP method compare with traditional simulation method. The Figure is drawn by Microsoft Office 2019. The figures of the Step 1 and Step 2 are drawn by Microsoft Office 2019. The figure of the Step 1–2 is from publicly available platforms on the Internet. The figure of the Step 1–3 is from ITTF website (https://cn.ittf.com/). The figure of the Step 3 and Step 4 are drawn by Python 3.8.8 (https://www.python.org/) and Microsoft Office 2019 (https://www.microsoft.com/).

The study design is shown in Fig. 1.

### Data resources

All videos are taken from publicly available platforms on the Internet. 100 international men’s singles table tennis competitions from 2019 to 2022, with a total of 8535 rallies are analyzed, including 59 elite male players. There are 200 data in the dataset (training data = 160, testing data = 40), each with 28 input values and 1 output value. Meanwhile, the technical styles (TS) are obtained from the International Table Tennis Federation website (https://www.ittf.com/), namely left-handed-penhold attackers (RPA), right-handed-penhold attacker (LPA), left-handed-shakehand attacker (LSA), right-handed-shakehand attacker (RSA), and right-handed-shakehand defender (RSD). The players’ singles winning rates (WR) from 2019 to 2022 are obtained from the International Table Tennis Federation website. They are calculated by filtering all matches from the players’ careers, excluding doubles and team matches.

### Data reliability

The intraclass correlation coefficient (ICC) is a statistical estimate that measures the extent of agreement between at least two quantitative measurements^40^. In order to test the validity of data collection and match analysis, 10 matches are randomly selected for observation and reanalysis, and the ICC test is conducted using IBM SPSS Statistics 27. The model and type of the ICC test are a 2-way mixed-effects model and absolute agreement, where the results of the single rater are 0.944 and 0.995, indicating excellent agreement^41^.

### Data clustering

K-means clustering is performed to classify the different levels of players’ singles WRs via Python 3.8 and the scikit-learn library. Before K-means clustering, this study adopted the Min–Max scaling for the players’ singles WRs. Comparing the performance of the binary, triple and four classifications, the best is the triple classification (high, medium, and low), and its silhouette coefficient is 0.604, as shown in Table 1.

**Table 1:** The results of the K-means clustering.

Clusters	The mean of silhouette coefficient	Labels & numbers
2	0.598181	0: 36; 1: 23
3	0.604239	0: 12; 1: 20; 2: 27
4	0.577663	0: 13; 1: 10; 2: 26; 3: 10

### Data formatting and normalization

In feature coding, label encoding is used to encode the three levels of players’ singles WRs (high: 3; medium: 2; low: 1), and the frequency encoding is used to encode the five technical styles (RPA: 11; RSA: 128; RSD: 10, LPA: 18, LSA: 33), which is consistent with previous studies in the data encoding^42–44^. In the process of data normalization, the study uses the Min–Max scaling.

### Traditional three-phase evaluation theory

The traditional three-phase evaluation theory is one of the most representative theoretical approaches in table tennis tactical diagnosis^1^. It is based on the serial characteristics of the last stroke scored or lost in each rally and divides the match into three phases: the serve-attack phase, the receive-attack phase, and the stalemate phase. The criteria for each phase are as follows: (1) The serve-attack phase: the points won and lost by the player on the serve (first stroke) and the third stroke, and the points lost on the fifth stroke; (2) The receive-attack phase: the points scored and lost by the player on the receive-attack phase (second stroke) and the fourth stroke. (3) The stalemate phase: the serving player scores on the fifth stroke and the scores lost points after the fifth stroke.

### Match progress phase in the double three-phase evaluation theory

As the rules of table tennis continue to evolve and the equipment on the table tennis court continues to change, the original theory of technical–tactical analysis may need to develop accordingly. Dr Dandan Xiao proposed the double three-phase evaluation theory^7^, adding the concept of match progression based on the traditional three-phase evaluation theory of the serve-attack phase, the receive-attack phase, and the stalemate phase, and classifies the whole match into the beginning phase, the middle phase, and the end phase. It is a more comprehensive and concise statistical method to analyze matches. The criteria for each phase are as follows: (1) the beginning phase: from the start of a game until one player scores four points; (2) the middle phase: from the time one player scores four points until eight points; (3) the end phase: from the time one player scores eight points until the end of the match.

### Stroke classification in the four-phase evaluation theory

Many studies use the traditional three-phase evaluation theory with great success. However, one problem has arisen in the long-term application the technical–tactical data of players on both sides of the match are unequal. In order to solve this problem and further optimize it, the four-phase evaluation theory is proposed to provide a complementary and symmetrical relationship between the two sides in each technical–tactical phase^3^. It can produce the matching data between two players and more detailed information on the stalemate phase. The four-phase evaluation theory classifies the whole match into four phases. These phases follow the criteria: (1) the serve-attack phase: the points won and lost at the first stroke and third strokes, as well as the points lost at the fifth strokes; (2) the receive-attack phase: the points won and lost at the second and fourth strokes; (3) the stalemate I phase: the points won at the fifth stroke, as well as the points, scored and lost after the fifth stroke; (4) the stalemate II phase: the points won and lost on the sixth stroke and beyond.

### Observation points

The observation points are based on the number of strokes and the last stroke scored to describe the profile of the table tennis match. It allows data to be collected quickly and more easily understood. The traditional three-phase evaluation theory and its various derivatives (the four-phase evaluation theory and the double three-phase evaluation theory) are proposed based on such an observation criterion for data collection. The observation points are as follows: (1) the odd number of strokes for the serving player (first, third, fifth, and beyond); (2) the even number of strokes for the receiving player (second, fourth, sixth, and beyond); (3) the last stroke won or lost by both players in each rally.

### Hybrid technical–tactical analysis theory

The double three-phase evaluation theory adds the concept of match progression to the traditional three-phase evaluation theory to analyze the match of table tennis in a multi-dimensional way^7^. However, it still follows the principles of the traditional three-phase evaluation theory for stroke classification in each phase, which indirectly retains the disadvantage that causes the mismatch of data between two players. Thus, it reflects that the usage rates (UR) in the corresponding phase of both players are not equal, and the sum of two scoring rates (SR) values is not 1, which is disadvantageous to the interactive analysis between both sides in the match. Nevertheless, the four-phase evaluation theory can better address the above issue^3^. However, the four-phase evaluation theory does not have the advantage that reflects the match progression proposed in the double three-phase evaluation theory. In order to provide an interactive and multi-perspective analysis of the match between both sides, this study hybridizes the advantages of two analysis theories into one hybrid technical–tactical analysis theory.

**Figure 2:**
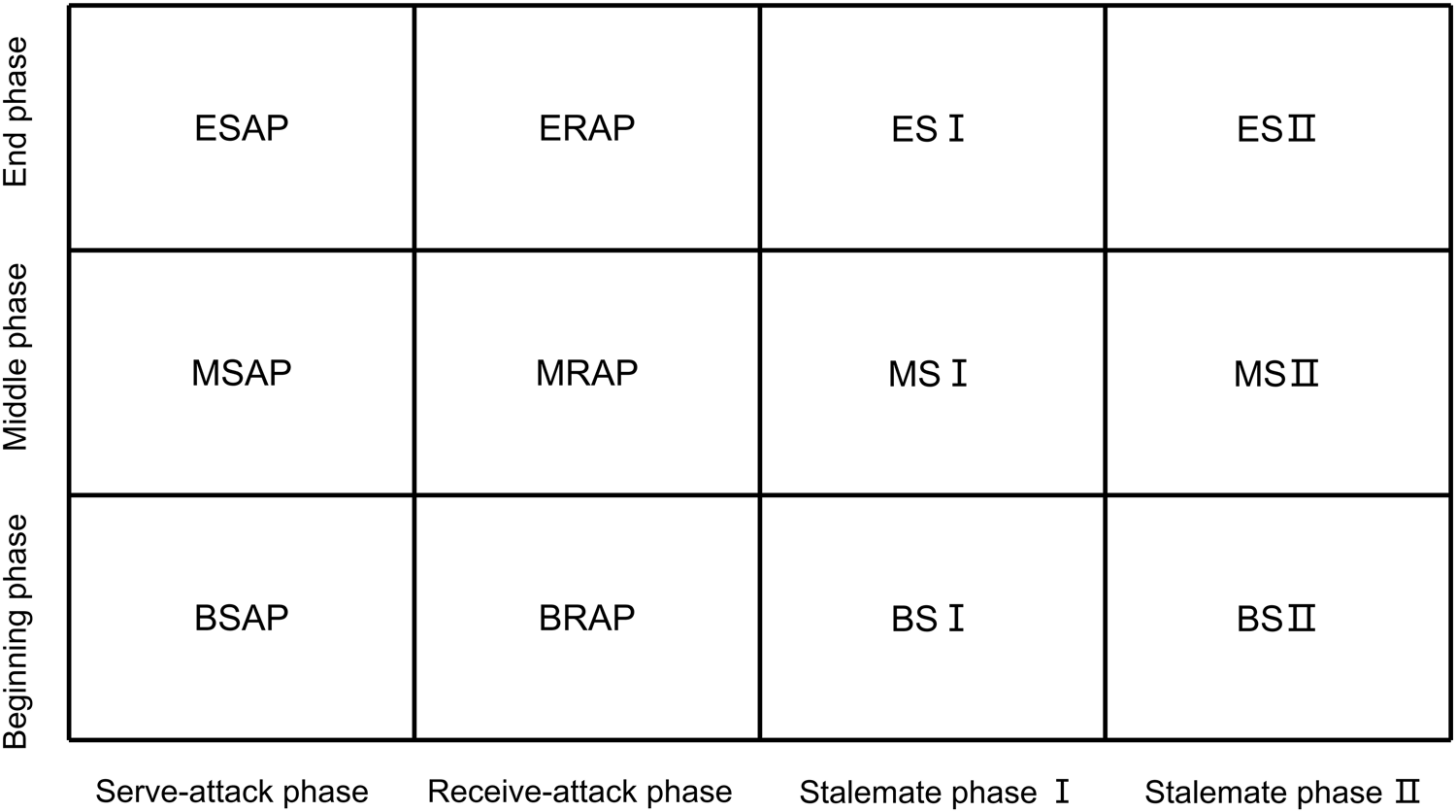
The framework of the hybrid technical–tactical analysis theory.

This study proposes a hybrid analysis theory by combining the match progression phase in the double three-phase evaluation theory and the four-phase evaluation theory for stroke classification for a total of twelve phases: the beginning and serve-attack phase (BSAP), the middle and serve-attack phase (MSAP), the end and serve-attack phase (ESAP), the beginning and receive-attack phase (BRAP), the middle and receive-attack phase (MRAP), the end and receive-attack phase (ERAP), the beginning and stalemate I phase (BSI), the middle and stalemate I phase (MSI), the end and stalemate I phase (ESI), the beginning and stalemate II phase (BSII), the middle and stalemate II phase (MSII), and the end and stalemate II phase (ESII). The framework of the hybrid technical–tactical analysis theory is shown in Table 2 and Fig. 2.

**Table 2:** The framework of the hybrid technical–tactical analysis theory.

	Beginning phase (point 0–4)	Middle phase (point 4–8)	End phase (point 8–end)	Situations	Observation points	
Serve-attack phase	1st, 3rd	1st, 3rd	1st, 3rd	Scored, lost	The odd number of strokes for the serving player	The last stroke scored or lost by both players
	5th	5th	5th	Lost		
Stalemate I phase	5th	5th	5th	Scored, lost		
	> 5th	> 5th	> 5th	Lost		
Receive-attack phase	2nd, 4th	2nd, 4th	2nd, 4th	Scored, lost	The even number of strokes for the receiving player	
Stalemate II phase	≧ 6th	≧ 6th	≧ 6th	Scored, lost

### Evaluation indicators

UR and SR are important indicators for evaluating a table tennis player’s performance. Whether it is the traditional three-phase evaluation theory, the double three-phase evaluation theory, or the four-phase evaluation theory, each of these phases’ indicators can be calculated by SR and UR, which are calculated as follows:

1 $${\text{SR}} = {\frac{{\text{phase }}\;{\text{score}}}{{\text{phase }}\;{\text{score }} + {\text{ phase}}\;{\text{points}}\;{\text{lost}}}\times 100\%}$$

2 $$\text{UR} = {\frac{\text{phase } \; \text{score } + \text{ phase } \; \text{points } \; \text{lost}} {\text{points} \; \text{scored} \; \text{and} \; \text{lost} \; \text{in} \; \text{the} \; \text{match} \; \text{progression}} }\times 100\%$$

The SR is expressed as phase score/(phase score + phase points lost) * 100%, and the UR is calculated as (phase score + phase points lost)/points scored and lost in the match progression (i.e., beginning, middle, or end).

As shown in Eq. (3), to further construct the link between SR and UR, the technique effectiveness (TE) is proposed to evaluate their contribution to the match results, which can help us better understand the effectiveness of players’ performances in each match phase^45^. Therefore, this study constructs a linkage between SR and UR through TE. Subsequently, the TEs of each phase are used as feature input to the model. TE ensures the interrelationship between SR and UR within each phase and considers the non-linear relationship between each phase.

3 $${\text{TE}} = - \left( {1 + \frac{{\sqrt 2 }}{2}} \right) + \left( {1.5 + \sqrt 2 } \right)\left[ {\left( {1 + {\text{UR}}} \right)^{{{\text{SR}} - 0.5}} } \right] - \frac{{\sqrt 2 }}{2}\left[ {\left( {1 + {\text{UR}}} \right)^{{2\left( {{\text{SR}} - 0.5} \right)}} } \right]$$

The overall winning probability (WP) indicates one player’s scoring ability. The mutual winning probability indicates the athlete’s scoring ability in a confrontational situation. One player’s overall winning probability (WP) is the total points scored/(total points scored + total points lost), as shown in Eq. (4). Moreover, the mutual winning probability (MWP) is ${\mathrm{WP}}_{\mathrm{player A}}$ − ${\mathrm{WP}}_{\mathrm{player B}}$, referring to the winning probability of player A over that of player B, as shown in Eq. (5).

4 $${\text{WP}} = \frac{{{\text{player}}\;{\text{A}}\;{\text{total}}\;{\text{points}}\;{\text{scored }}}}{{{\text{total}}\;{\text{points}}\;{\text{scored }} + {\text{ total}}\;{\text{points}}\;{\text{lost}}}}$$

5 $${\text{MWP}} = {\text{WP}}_{{{\text{player}}\;{\text{A}}}} - {\text{WP}}_{{{\text{player}}\;{\text{B}}}}$$

### KNN

The k-nearest neighbour (KNN) algorithm is a supervised learning algorithm for classification and regression problems. It is easy to implement and does not rely on any specific model, making it simple and popular. The algorithm examines the k nearest data to the training set to classify new samples. These k examples belong to a large class to which this new sample belongs^46^. KNN is computed by the preferred method of Euclid’s formula, where the mathematical expression can be seenin Eq. (6).

6 $${\text{d}} = \sqrt {\mathop \sum \limits_{{{\text{i}} = 1}}^{{\text{n}}} \left( {{\text{x}}_{{\text{i}}} - {\text{y}}_{{\text{i}}} } \right)^{2} }$$

### DT

The Decision Tree (DT) is a non-parametric supervised learning algorithm. Classification and Regression Trees (CART) type of DT is used in this study to go for the regression task. It is a hierarchical tree structure consisting of a root node, branches, internal nodes and leaf nodes.

### RF

The Random Forest (RF) is a fast and robust integrated learning method first described by Breiman^47^ that can be used for classification and regression. RF regression creates multiple decision trees from bootstrap samples of the training dataset, and the average of the N outputs of the N decision trees is calculated by voting to create the final Projection^48^, where the final mathematical expression is shown in Eq. (7).

7 $$\hat{f}_{RF}^{C} \left( x \right) = \frac{1}{C}\mathop \sum \limits_{i = 1}^{C} T_{i} \left( x \right)$$

where *x* is the vector input variable, *C* is the number of trees, and ${T}_{i}(x)$ is a single regression tree constructed based on a subset of the input variables and the bootstrapped samples.

### ET

The extra trees algorithm (ET) is a relatively new machine learning technique developed as an extension of the Random Forest algorithm, using the same principles as Random Forest and using a random subset of features to train each base estimator and the ability to randomly select the best features and corresponding values to segment the nodes.

### XGB

The eXtreme Gradient Boosting (XGB) algorithm, a scalable tree augmentation system proposed by Dr Chen, is now widely used by data scientists and provides state-of-the-art results on many problems^49^. Given the training data ${\left\{\left({x}_{i},{y}_{i}\right)\right\}}_{i=1}^{N}$, the objective function of XGBoost consisting of two parts reads:

8 $${\text{Obj}} = \mathop \sum \limits_{i = 1}^{n} {\text{l}}\left( {{\text{y}}_{{\text{i}}} ,{\hat{\text{y}}}_{{\text{i}}} } \right) + \mathop \sum \limits_{k = 1}^{n} {\Omega }\left( {{\text{f}}_{{\text{k}}} } \right)$$

where Obj is the minimization objective, $\mathrm{l}\left({\mathrm{y}}_{\mathrm{i}},{\widehat{\mathrm{y}}}_{\mathrm{i}}\right)$ is the loss function for each accident sample, and it measures the error between the actual value and the predicted value. $\Omega \left({\mathrm{f}}_{\mathrm{k}}\right)$ is the conventional term used to prevent overfitting.

Another significant enhancement of the XGB algorithm is the ability to optimize the objective function using both the first-order derivative and the second-order derivative of a function of sorts:

9 $${\text{Gain}} = \frac{1}{2}\left[ {\frac{{\left( {\mathop \sum \nolimits_{{{\text{i}} \in {\text{I}}_{{\text{L}}} }} {\text{g}}_{{\text{i}}} } \right)^{2} }}{{\mathop \sum \nolimits_{{{\text{i}} \in {\text{I}}_{{\text{L}}} }} {\text{h}}_{{\text{i}}} + {\uplambda }}} + \frac{{\left( {\mathop \sum \nolimits_{{{\text{i}} \in {\text{I}}_{{\text{R}}} }} {\text{g}}_{{\text{i}}} } \right)^{2} }}{{\mathop \sum \nolimits_{{{\text{i}} \in {\text{I}}_{{\text{R}}} }} {\text{h}}_{{\text{i}}} + {\uplambda }}} - \frac{{\left( {\mathop \sum \nolimits_{{{\text{i}} \in {\text{I}}}} {\text{g}}_{{\text{i}}} } \right)^{2} }}{{\mathop \sum \nolimits_{{{\text{i}} \in {\text{I}}}} {\text{h}}_{{\text{i}}} + {\uplambda }}}} \right]$$

where ${g}_{i}$ denotes the first and second-order partial derivatives of the loss function of the previous tree model, ${\mathrm{I}}_{\mathrm{L}}$ and ${\mathrm{I}}_{\mathrm{R}}$ respectively denote the set of samples in the left and right nodes; in addition, $\mathrm{I}={\mathrm{I}}_{\mathrm{L}}{\mathrm{UI}}_{\mathrm{R}}$

### BPNN

Back Propagation Neural Network (BPNN) is also a prevalent neural network technique for engineering applications, which is a multi-layer artificial neural network including an input layer, hidden layer and output layer^50^. The BPNN algorithm minimizes the mean square error between the predicted and desired outputs. For a set of input data x(t) and output data y(t), the mapping relationship between them can be obtained by using BPNN models as follows:

10 $$y_{k}^{t + T} = \mathop \sum \limits_{j = 1}^{M} f_{2} \left[ {w_{jk} f_{1} \left( {\mathop \sum \limits_{i = 1}^{N} w_{ij} x_{i}^{t} - \theta_{j} } \right) - \theta_{k} } \right]$$

where ${x}_{i}^{t}$ is the input value of *i*th node at the time of t, ${w}_{ij}$ is the weight value between the* i*th node of input layer and the *j*th node of hidden layer, ${\theta }_{j}$ is threshold value at the *j*th node of hidden layer, ${w}_{jk}$ is the weight value between the *j*th node of hidden layer and the kth node of output layer, ${\theta }_{k}$ is threshold value at the kth node of output layer, ${y}_{k}^{t+T}$ is the output value at the* k*th node at the time of *t* + *T*, and* T* is the forecast period. ${f}_{1}$(·) and ${f}_{2}$(·) are activation functions.

### LSTM and LSTM–BPNN

**Figure 3:**
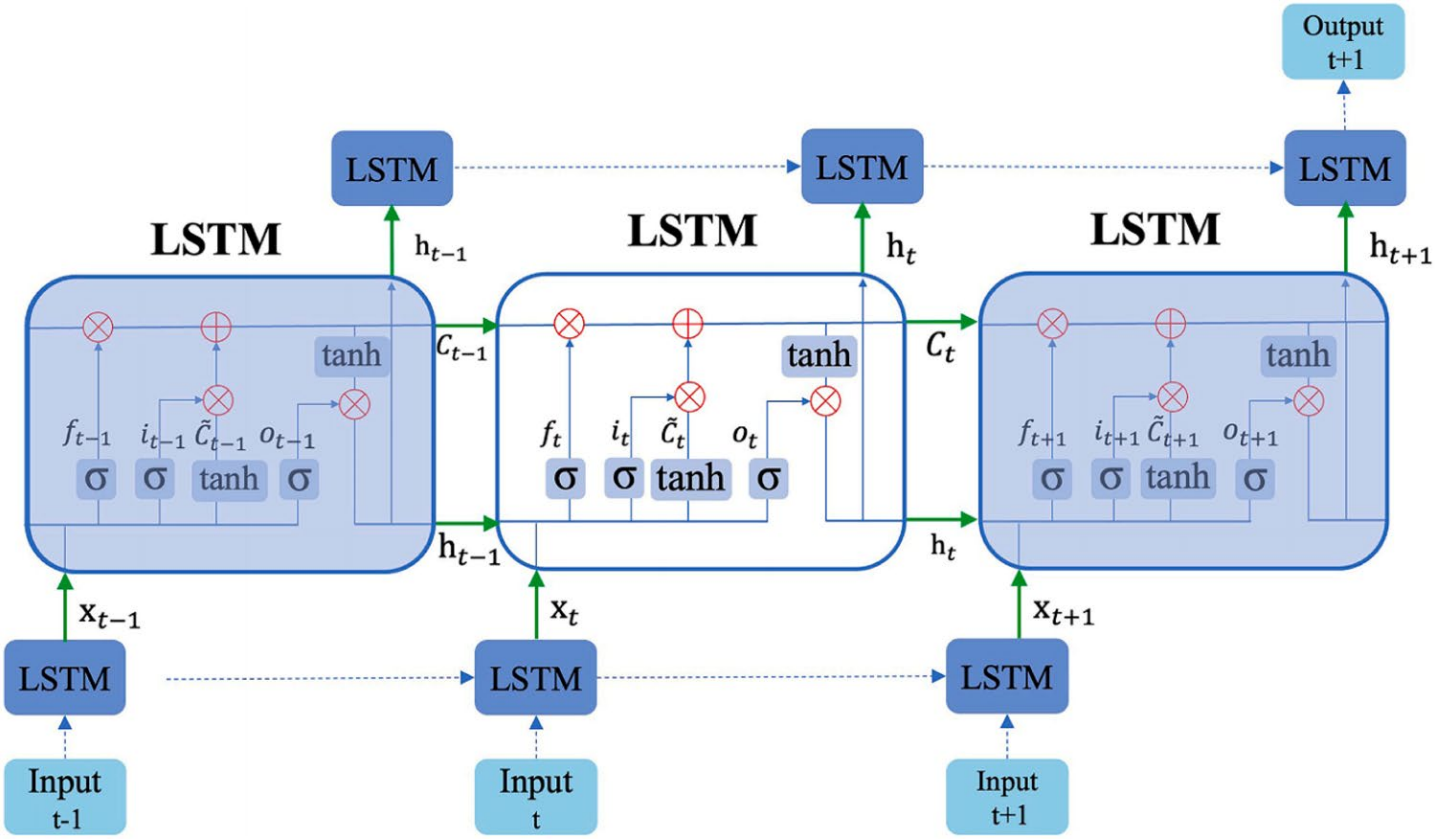
The framework of the long short-term memory neural network^51^.

The long short-term memory neural network (LSTM) was proposed by Hochreiter et al. In order to eliminate the gradient disappearance problem^30^, unlike the general RNN, which consists of three types of layers: output, hidden and input layers, the LSTM consists of three gates (forgetting gate, input gate, output gate) and additional cell memory, as shown in Eqs. (11–18) and Fig. 3. The forgetting gate determines what information is forgotten from the previous state, the inputs determine what information is updated and stored in the previous state, and the output gate determines the final output of the information. The equations in the LSTM at the t-th layer are summarized as follows, t = 0, …, τ.

11 $${\text{Forget gate}} {:}\;{\text{f}}_{{\text{t}}} =\upsigma \left( {{\text{x}}_{{\text{t}}} {\text{W}}_{{\text{f}}} + {\text{h}}_{{{\text{t}} - 1}} {\text{W}}_{{\text{f}}} + {\text{b}}_{{\text{f}}} } \right)$$

12 $${\text{Input gate }}{:}\;{\text{ i}}_{{\text{t}}} = {\upsigma }\left( {{\text{x}}_{{\text{t}}} {\text{W}}_{{\text{i}}} + {\text{h}}_{{{\text{t}} - 1}} {\text{W}}_{{\text{i}}} + {\text{b}}_{{\text{i}}} } \right)$$

13 $$\mathrm{Old \;value}{:}\; {\widehat{\mathrm{C}}}_{\mathrm{t}}=\mathrm{tan}\;\mathrm{h}\left({\mathrm{x}}_{\mathrm{t}}{\mathrm{W}}_{\mathrm{c}}+{\mathrm{h}}_{\mathrm{t}-1}{\mathrm{W}}_{\mathrm{c}}+{\mathrm{b}}_{\mathrm{c}}\right)$$

14 $${\text{New cell state }}{:}\;{\text{ C}}_{{\text{t}}} = {\text{f}}_{{\text{t}}} \odot {\text{C}}_{{{\text{t}} - 1}} + {\text{i}}_{{\text{t}}} \odot {\tilde{\text{C}}}$$

15 $${\text{Output gate }}{:}\;{\text{ o}}_{{\text{t}}} = {\upsigma }\left( {{\text{x}}_{{\text{t}}} {\text{W}}_{{\text{o}}} + {\text{h}}_{{{\text{t}} - 1}} {\text{W}}_{{\text{o}}} + {\text{b}}_{{\text{o}}} } \right)$$

16 $${\text{Hidden state }}{:}\;{\text{ h}}_{{\text{t}}} = {\text{o}}_{{\text{t}}} \odot \tan \left( {{\text{C}}_{{\text{t}}} } \right)$$

17 $${\text{S}} {-} {\text{type function}}{:}\;\upsigma \left( {\text{z}} \right) = \frac{1}{{1 + {\text{e}}^{{ - {\text{z}}}} }}$$

18 $${\text{Tanh}} {-} {\text{type function}}{:}\; {\text{tanh}}\left( z \right) = \frac{{e^{z} - e^{ - z} }}{{e^{z} + e^{ - z} }}$$

where *W* is the weight matrices, *b* is the bias vectors, step length or size *t* > 0 and $\odot$ denotes the Hadamard product.

**Figure 4:**
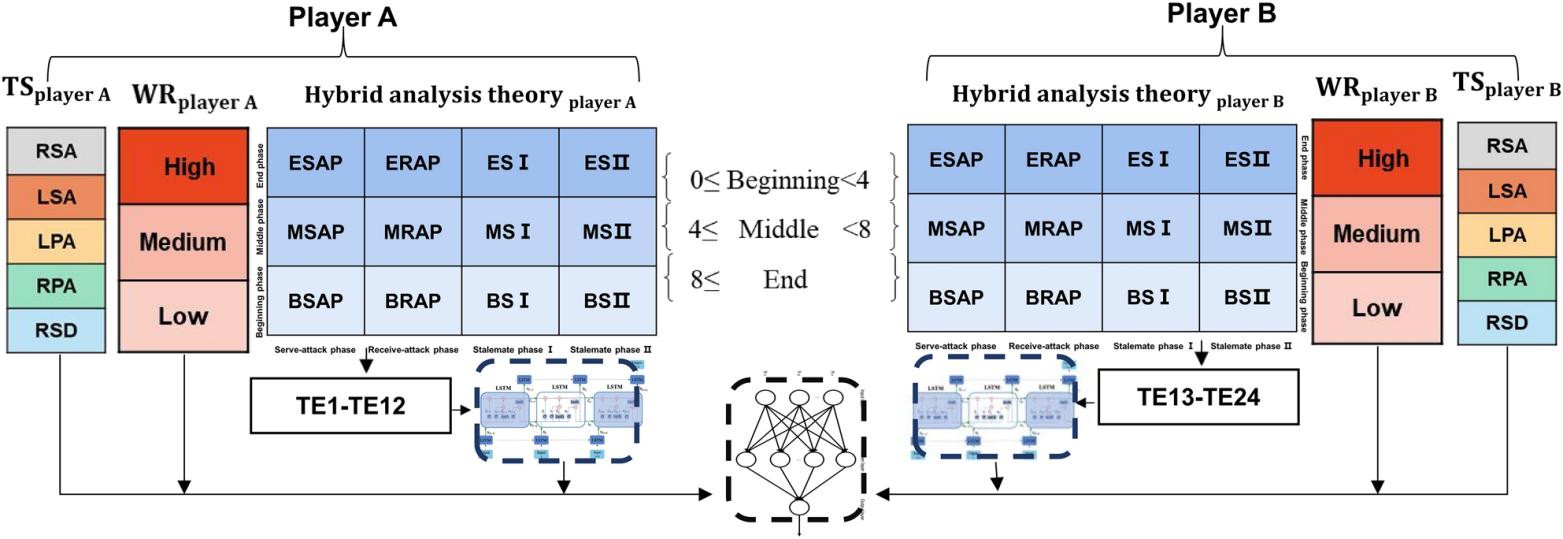
The framework of the interactive technical–tactical model based on LSTM–BPNN diagnosis model.

This study proposes a hybrid algorithm, namely LSTM–BPNN, and utilizes LSTM to extract features of the serial relation of each technical–tactical phase in the match. After feature extraction of both sides, the BPNN is used for non-linear regression. The specific framework of the model is shown in Supplementary file and Fig. 4. KNN, DT, RF, ET, and XGB modelling are done via the sci-kit-learn library in Python 3.8. BPNN and LSTM–BPNN modelling is done via the Pytorch library in Python 3.8.

### SHAP

**Figure 5:**
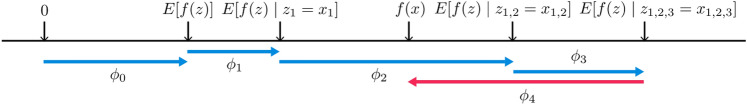
SHAP attributes^35^.

SHAP is an additive explanatory model based on cooperative game theory^52^, which defines the output model as a linear sum of the input models. In SHAP, all feature values in the sample are considered as “contributors” to the prediction machine learning model, and for each prediction sample, SHAP can output a unique solution as the evaluation value of the sample features, i.e., the SHAP value^35^. Based on the additive feature attribution methods, SHAP constructs a linear function g with binary variables to estimate the target function f. The explanation model is as follows:

19 $${\text{F}}\left( {\text{x}} \right) \approx {\text{g}}\left( {{\text{z}}^{\prime } } \right) = \phi_{0} + \mathop \sum \limits_{{{\text{i}} = 1}}^{{\text{M}}} \phi_{{\text{i}}} {\text{z}}_{{\text{i}}}^{\prime }$$

where ${z}^{^{\prime}}\approx {x}^{^{\prime}}$, where ${x}^{^{\prime}}$ is the simplified input that mapped from the original inputs $x={h}_{x}({x}^{^{\prime}})$ and ensure that $g({\mathrm{z}}^{\mathrm{^{\prime}}})\approx f({h}_{x}({x}^{^{\prime}}))$; ${\phi }_{0}$ is the contribution with zero inputs, and *M* is the number of simplified input features. Equation (19) is illustrated in Fig. 5.

As noted by Lundberg and Lee^35^, a single solution exists for Eq. (19), which has three desirable properties: local accuracy, missingness, and consistency. The local accuracy indicates that for any specific data *x*, the output of the explainable model *g* shall match the output of the original model *f* when x = ${h}_{x}$(${x}^{i}$), i.e. $f(x) = g({x}^{i})$. Missingness indicates that there shall be no impact on contribution for the features that are missing in the simplified inputs, i.e. ${x}_{i}^{^{\prime}}=0$, implying ${\phi }_{i} = 0$. Consistency implies that the inputs attribution should not be changed even though the simplified inputs’ contribution varies due to the change in the model^53^. Specifically, for any two models *f* and ${f}^{i}$, if ${f}_{x}^{^{\prime}}({\mathrm{z}}^{\mathrm{^{\prime}}})-{f}_{x}^{^{\prime}}\left({z}^{^{\prime}}\backslash i\right)\ge {f}_{\mathrm{x}}\left({z}^{^{\prime}}\right)-{f}_{\mathrm{x}}\left({z}^{^{\prime}}\backslash i\right), then \; {\phi }_{i}({f}^{^{\prime}}, x)\ge {\phi }_{i}(f, x)$, where ${z}^{^{\prime}}\backslash i$ indicates that input without ${z}_{i}^{^{\prime}}$. Consequently, the only possible model that satisfies these properties is Eq. (20).

20 $$\phi_{i} \left( {{\text{f}},{\text{x}}} \right) = \mathop \sum \limits_{{{\text{z}}^{\prime } \subseteq x^{\prime}}} \frac{{\left| {z^{\prime}} \right|!\left( {{\text{M}} - \left| {z^{\prime}} \right| - 1} \right)!}}{{{\text{M}}!}}\left[ {{\text{f}}_{{\text{x}}} \left( {z^{\prime}} \right) - {\text{f}}_{{\text{x}}} \left( {z^{\prime}\backslash i} \right)} \right]$$

where $\left|{z}^{^{\prime}}\right|$ is the number of non-zero entries in ${z}^{^{\prime}}$, and ${\phi }_{i}$ is the Shapley value for feature ${x}_{i}$.

### Traditional technical–tactical diagnosis based on simulation method

Two players’ TEs in each phase and their mutual correlations are shown in Table 3. Technical–tactical Eq. (21) is proposed to calculate one player’s magnitude of increasing or decreasing the SR at one phase, with other indicator values unchanged^17,21^ for both players’ technical–tactical diagnosis in matches. If the SR is less than or equal to 50%, the incremental value Z = SR + Y; otherwise, the incremental value Z = SR − Y^17^. Subsequently, this player’s recalculated SR at one phase is used to calculate the new SR value of the other-side player at the corresponding phase by calculating the 1-SR formula and keeping each indicator value of other phases unchanged. Moreover, the recalculated SRs with the original URs of both sides are then applied through Eq. (3) to calculate the adjusted TEs. These two adjusted TEs and the other unchanged TEs are combined and re-input into the model to output the adjusted mutual winning probability (AMWP). The weight value is calculated via Eq. (22). The greater the weight’s absolute value is, the greater the influence of the adjusted phase on the MWP of both sides is and the more significant the impact on whether the player can win the match.

21 $${{\text{Y}} = 0.238 }{ \times } \cos \left( {{ - 1.32}{ \times }{\text{SR}} + 0.66} \right) - 0.178$$

22 $${\text{Weight}} = \frac{{\left| {{\text{MWP}} - {\text{AMWP}}} \right|}}{{{\text{MWP}}}}{ \times }100\%$$

**Table 3:** Two players’ technique effectiveness indicators in each phase and their mutual correlations.

Player A		Player B		Player A & Player B	
Phases	Variables	Phases	Variables	Phases	Variables
BSAP	TE1	BRAP	TE14	BSAP-BRAP	TE1&TE14
BRAP	TE2	BSAP	TE13	BRAP-BSAP	TE2&TE13
BSI	TE3	BS II	TE16	BSI-BSII	TE3&TE16
BSII	TE4	BSI	TE15	BS II -BSI	TE4&TE15
MSAP	TE5	MRAP	TE18	MSAP-MRAP	TE5&TE18
MRAP	TE6	MSAP	TE17	MRAP-MSAP	TE6&TE17
MSI	TE7	MSII	TE20	MSI-MSII	TE7&TE20
MSII	TE8	MSI	TE19	MSII -MSI	TE8&TE19
ESAP	TE9	ERAP	TE22	ESAP-ERAP	TE9&TE22
ERAP	TE10	ESAP	TE21	ERAP-ESAP	TE10&TE21
ESI	TE11	ESII	TE24	ESI-ESII	TE11&TE24
ESII	TE12	ESI	TE23	ESII -ESI	TE12&TE23

*TE* technique effectiveness.

### Technical–tactical diagnosis based on SHAP method

Unlike traditional table tennis tactical diagnostic methods that indirectly analyze the players’ technical–tactical performance by simulation, current study interprets the model by constructing an artificial intelligence model and using the state-of-the-art SHAP method, which assigns Shapley values to all table tennis technical–tactical indicators and can make a global analysis of the interaction of each indicator for the game. Unlike previous tree-based machine learning models that only present the feature importance, it is noteworthy that the SHAP value also reflects the positive and negative trends of feature importance on the model. In addition, the SHAP model calculates feature importance based on only one or local observations^53^, allowing us to focus our analysis on a specific player and a specific game, bringing table tennis technical–tactical diagnosis to a more precise level. Finally, because of the non-linear characteristics of the various influencing factors and indicators in table tennis competitions, traditional research methods only provide a rough interpretation of the game profile through feature weight values, while based on the SHAP method, we visualize the relationship between the independent and dependent variables and discuss the non-linear relationships between the influencing factors and indicators. Thus, the SHAP method used for table tennis technical–tactical diagnostic analysis can address the shortcomings of traditional methods and enhance the benefits of technical–tactical diagnosis.

## Result and discussion

### Performance comparison

In order to find the best algorithm for prediction accuracy, this study compares the performance of LSTM–BPNN with six classical artificial intelligence algorithms, which are BPNN, XGB, RF, ET, DT and KNN, in terms of the mean squared error (MSE), mean absolute error (MAE), root mean square error (RMSE), and coefficient of determination (${\mathrm{R}}^{2}$), as shown in Eqs. (23–26).

23 $${\text{R}}^{2} = 1 - { }\frac{{\mathop \sum \nolimits_{{{\text{i}} = 1}}^{{\text{n}}} \left( {{\text{y}}_{{\text{i}}} { } - {\hat{\text{y}}}_{{\text{i}}} } \right)^{2} }}{{\mathop \sum \nolimits_{{{\text{i}} = 1}}^{{\text{n}}} \left( {{\text{y}}_{{\text{i}}} { } - {\overline{\text{y}}}_{{}} } \right)^{2} }}$$

24 $${\text{MSE}} = \frac{1}{{\text{n}}}{ }\mathop \sum \limits_{{{\text{i}} = 1}}^{{\text{n}}} ({\hat{\text{y}}}_{{\text{i}}} - {\text{y}}_{{\text{i}}} )^{2}$$

25 $${\text{RMSE}} = \sqrt {\frac{1}{{\text{n}}}\mathop \sum \limits_{{{\text{i}} = 1}}^{{\text{n}}} \left( {{\text{y}}_{{\text{i}}} - {\hat{\text{y}}}_{{\text{i}}} } \right)^{2} }$$

26 $$\text{MAE} = {\frac{1}{\text{n}}}\mathop\sum\limits_{\text{i} = 1}^{\text{n}} | {\widehat{\text{y}}}_{\text{i}} - {\text{y}}_{\text{i}} |$$

${\mathrm{y}}_{\mathrm{i}}$: the original value; ${\widehat{\mathrm{y}}}_{\mathrm{i}}$: the simulated value; ${\overline{\mathrm{y}} }$: the sample mean; n: the number of samples.

This study uses the tenfold cross-validation to determine the hyper-parameters of XGB, RF, ET, DT, KNN, LSTM–BPNN, and BPNN for improving the model performance. The dataset is split and shuffled into the training set and testing set (training:testing, 8:2). The training set is split and shuffled as 10 consecutive folds. Each fold is treated as a validation set, and the remaining 9 folds as the training sets. The best hyper-parameters of XGB, RF, ET, DT, KNN, LSTM–BPNN, and BPNN depend on their performance by calculating the mean value of these ten validations.

The final hyper-parameters of LSTM–BPNN and BPNN are as follows: (1) LSTM–BPNN (1 LSTM layer with 50 hidden layer neurons, 40 BPNN hidden layer neurons, 0.01 learning rate and 200 epochs) applied the Adam as the optimizer and the MSELoss as the criterion; (2) BPNN (31 hidden neurons, 0.01 learning rate and 200 epochs) applied the Adam as the optimizer and the MSELoss as the criterion. The hyper-parameters of DT, ET, RF and KNN are as follows: (1) DT (criterion = ‘squared error’, adept = 2, min_samples_split = 2); (2) ET (n_estimators = 2500, max_depth = 15, min_samples_split = 2, min_samples_leaf = 1); (3) XGB (learning_rate = 1, max_depth = 1, min_child_weight = 4, n_estimators = 2500); (4) RF (n_estimators = 1000, min_samples_split = 2, max_depth = 15); (5) KNN (n_neighbors = 9).

Table 4 shows the testing results of LSTM–BPNN, BPNN, DTR, XGBR, RFR, KNN and ETR. The results show that LSTM–BPNN outperforms the others in testing data, with MSE = 0.000355, MAE = 0.014237, RMSE = 0.018853, and ${\mathrm{R}}^{2}$ = 0.988311. Therefore, LSTM–BPNN can provide more authentic feedback. Using LSTM–BPNN as the predicting model to calculate SHAP values for further interpretation and analysis is applicable and consistent with comparative studies^54^.

**Table 4:** Comparison of 7 artificial intelligence models.

	MSE	MAE	RMSE	${\mathrm{R}}^{2}$
LSTM–BPNN	0.000355	0.014237	0.018852	0.988311
BPNN	0.000560	0.017646	0.023672	0.981568
XGB	0.001964	0.036606	0.044312	0.935414
RF	0.004427	0.055414	0.066534	0.854396
ET	0.003561	0.050508	0.059671	0.882883
DT	0.017402	0.107233	0.131918	0.427607
KNN	0.008065	0.071411	0.089805	0.734728

### Global model interpretation with SHAP

Previous studies have shown that multiple factors influence the results of ball games, and the influencing factors show a non-linear relationship with each other^26,55–59^. This view is verified by related studies^17,21^, which all used artificial intelligence algorithms with the ability to handle nonlinearities. The result of this study is consistent with the design and viewpoints of the above studies. Although artificial intelligence algorithms have considerable accuracy, the lack of interpretation makes it difficult to understand the internal mechanism of the model and the relationship between individual features^60^. Therefore, the SHAP is adopted in this study to explain the technical–tactical diagnosis model based on LSTM–BPNN, which allows us to understand which features or phases are essential in the current table tennis matches.

**Figure 6:**
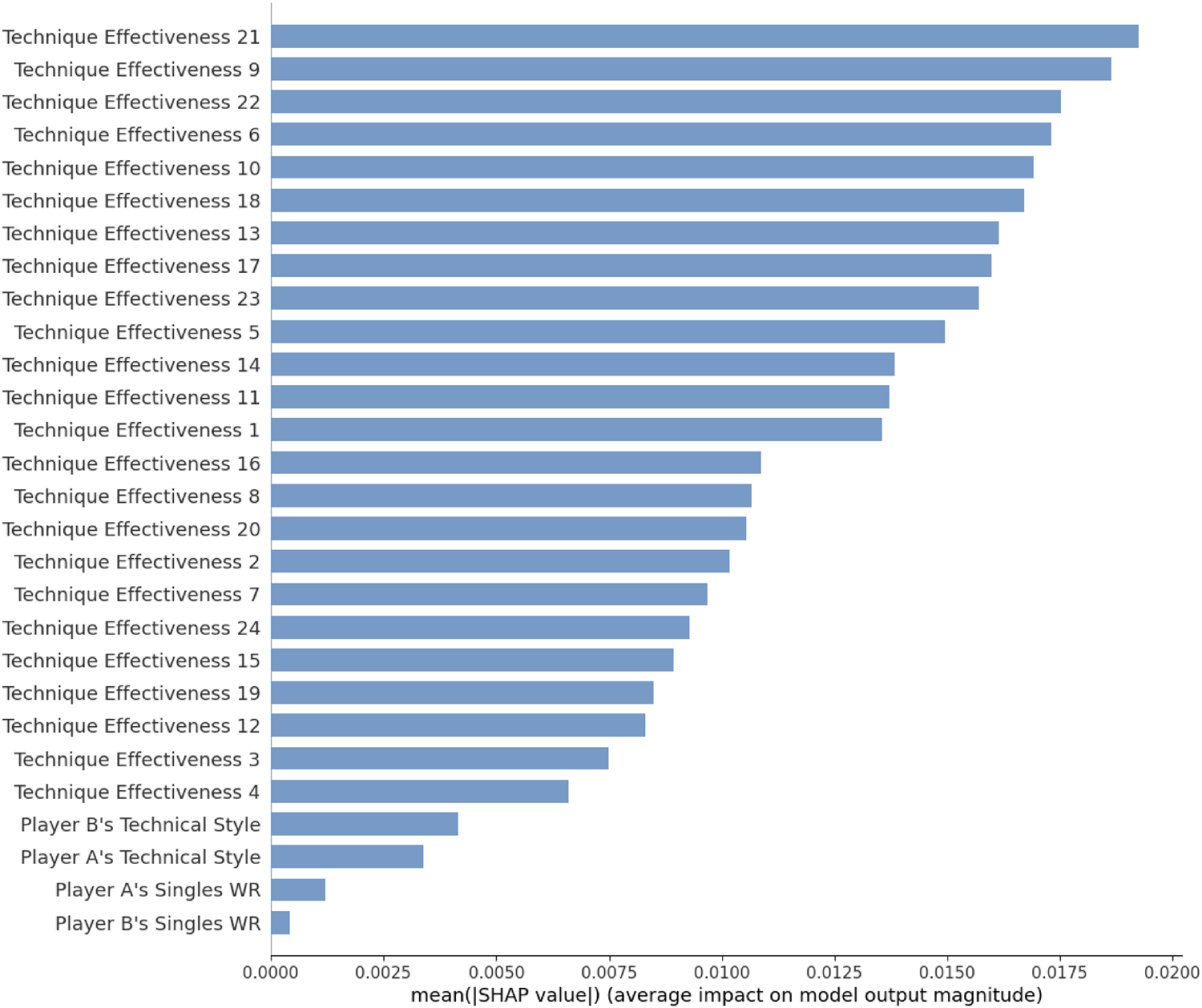
The LSTM–BPNN model’s Feature importance in terms of the mean magnitude of SHAP values. Figure is drawn by Python 3.8.8 (https://www.python.org/).

Figure 6 indicates the training dataset’s relative importance and shows the SHAP value’s mean magnitude applied to characterize the feature importance of the LSTM–BPNN model. The higher the feature’s importance is, the more contribution the feature provides in predicting the model. 28 features are sorted according to their importance, and TE21 ranks top, followed by TE9, TE22, TE6 and TE10. The results show that player B’s ESAP, player A’s ESAP, player B’s ERAP, player A’s MRAP and player A’s ERAP greatly impact current table tennis matches. It implies that the receive-attack and serve-attack phases of the ending match have an essential impact on MWP. At the same time, in terms of the mutual correlations between the phase of player A and player B (Table 3), the ESAP-ERAP phase of them has a high impact on MWP.

**Figure 7:**
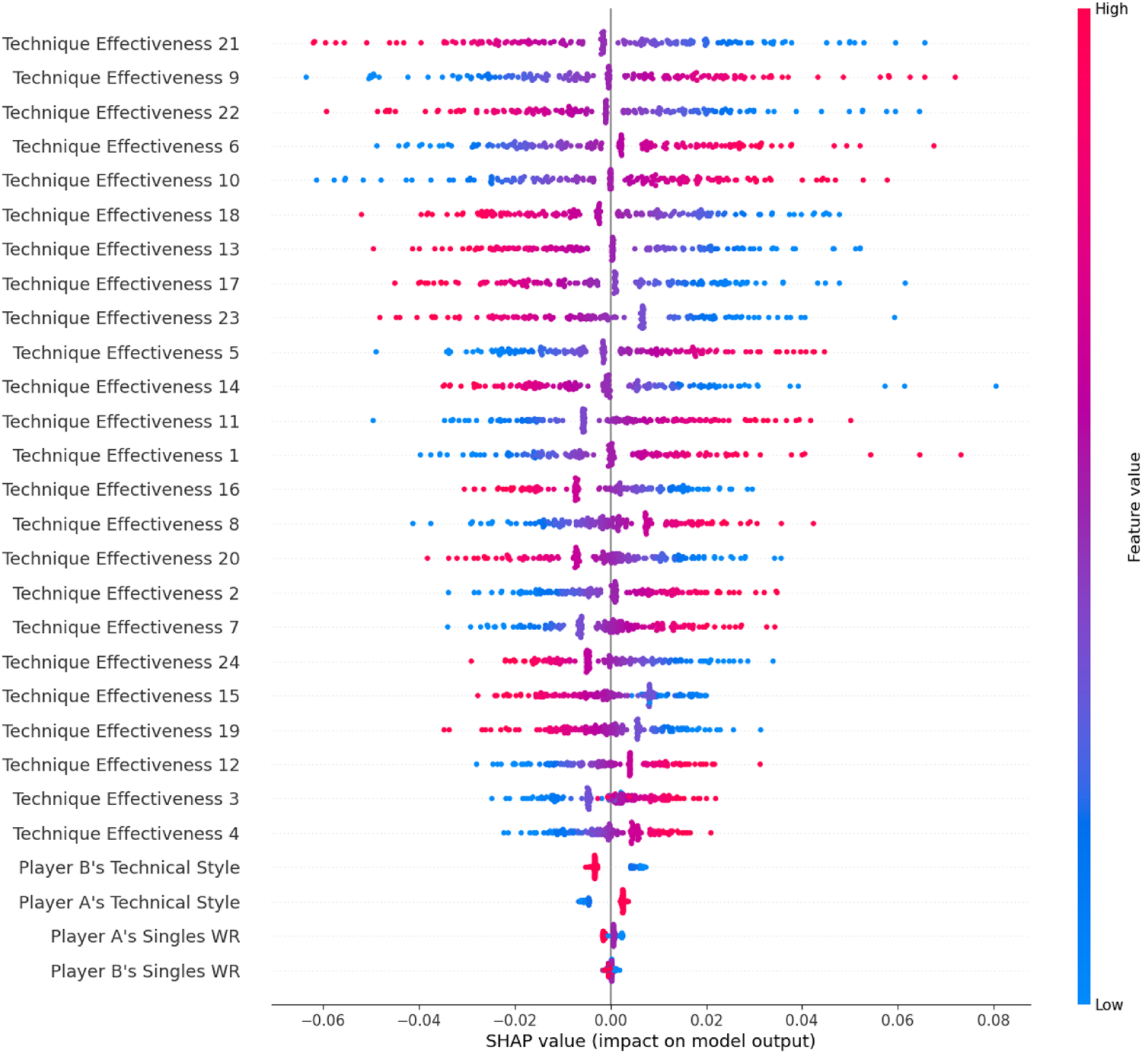
SHAP summary plot related to mutual winning probability. Figure is drawn by Python 3.8.8 (https://www.python.org/).

As shown in Fig. 7, the SHAP summary plots are provided to concisely describe the distribution of the feature’s impact on the MWP and the relationship between the features’ SHAP values and their impact. The dots with bluer colour refer to lower feature values. Figure 7 implies that the higher player A’s TEs are, the higher their SHAP values are, and the bigger MWPs are; the higher player B’s TEs are, the lower their SHAP values and the smaller MWPs are. Two players’ TEs exhibit mutually constraining impacts on the MWP in their corresponding phase (Table 3). Furthermore, high TEs can lead to low SHAP values for player B rather than for player A because of Eq. (5).

**Figure 8:**
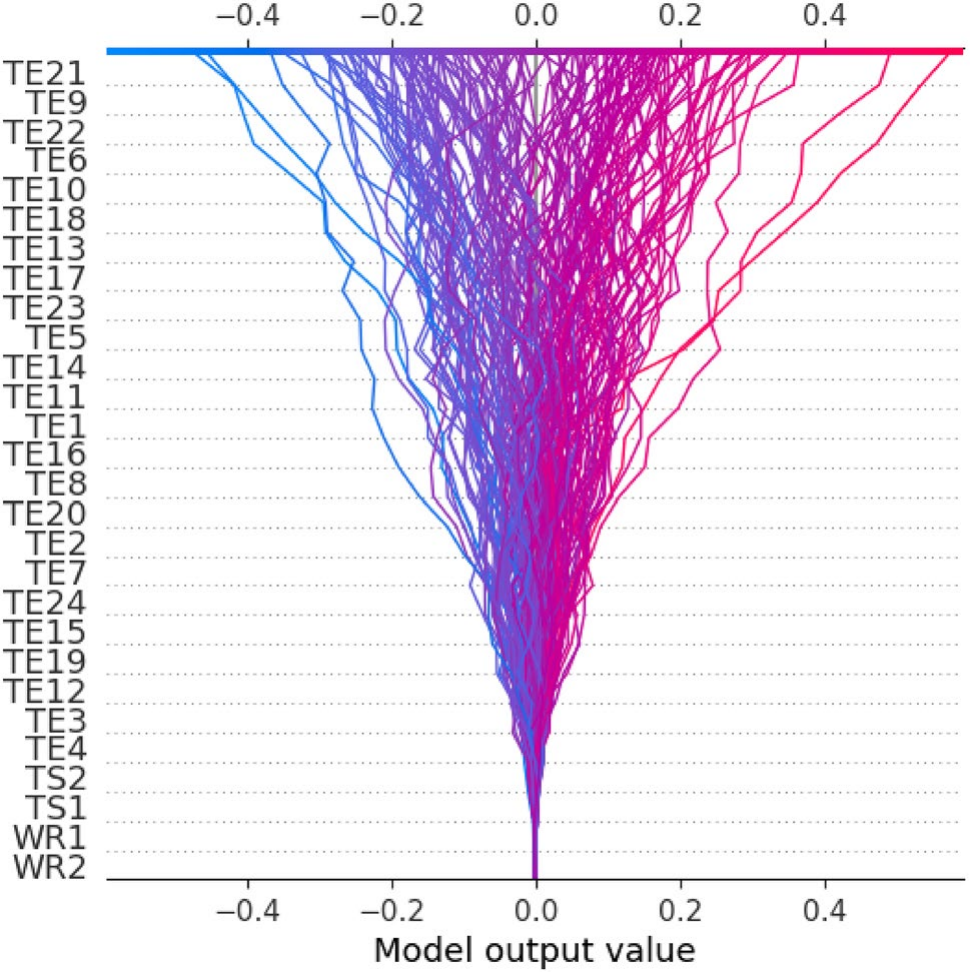
SHAP decision plot related to mutual winning probability. Figure is drawn by Python 3.8.8 (https://www.python.org/). Abbreviation: TE technique effectiveness; TS1 player A’s technical style; TS2 player B’s technical Style; WR1 player A’s singles winning rate; TS2 player B’s singles winning rate.

As shown in Fig. 8, the SHAP decision plot provides more detail on how each feature impacts the MWP in each data sample. To some extent, Fig. 8 implies that each feature contributes differently to the MWP in each data sample. It is consistent with the comparative studies that the quality of their opponents influences players’ performance, results, and rankings^61–65^.

**Figure 9:**
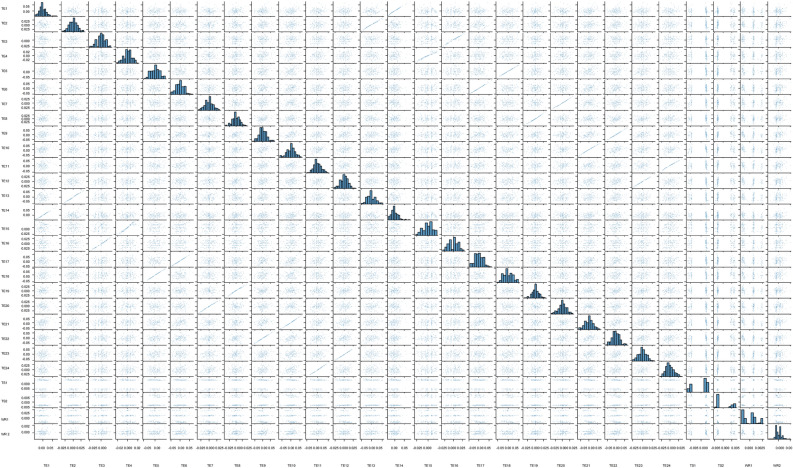
The relationship of features’ SHAP values. Figure is drawn by Python 3.8.8 (https://www.python.org/). Abbreviation: TE technique effectiveness; TS1 player A’s technical style; TS2 player B’s technical Style; WR1 player A’s singles winning rate; TS2 player B’s singles winning rate.

In order to further investigate whether these features’ SHAP values are linear or non-linear in the relationship, Fig. 9 is provided to describe this aspect concisely, and each TE’s SHAP value symbolizes the degree of impact on the final MWP. As shown in Fig. 9, the result finds that both players show an approximately linear relationship in each corresponding phase of the TEs’ SHAP values. The comparative studies showed that the phase-based analysis theory used two indicators, UR and SR, to better and more directly compare the technical–tactical performance of both players. Meanwhile, it reveals a near-complementary relationship between the SRs of both players at each corresponding phase, with URs presenting a balanced and equal relationship. TEs construct a non-linear relationship between URs and SRs. However, the TEs of both players also correspond to each corresponding phase, which results in the higher the TEs of one side, the lower the TEs of his opponent. The above result shows an approximately linear relationship. Furthermore, the result implies that the SHAP values of both players’ TEs, WRs and TSs show non-linear relationships, consistent with relative studies^66,67^. Meanwhile, the SHAP values of one side’s WR and TS have presented non-linear relationships with another side, consistent with relative studies^57,59^.

### Case diagnosis and local analysis

Regarding the technical–tactical diagnosis and analysis, SHAP provides the local interpretation to explain outputs, which is a different way to analyze and diagnose table tennis matches. Using SHAP reveals the essential feature in one match or the most crucial feature of one player in many matches. In the case application, as shown in Table 5, this study uses the traditional method and the SHAP method to analyze and diagnose the German elite male player Ovtcharov’s eight matches from 2019 to 2022 against Harimoto, Lin Yun-Ju, Morizono, Achanta, Alamian, Lin Gaoyuan, Lebesson, and Hirano.

**Table 5:** The specific information about Ovtcharov against eight players.

Opponents	Technical style	Winning rate	Won/lost
Harimoto	RSA	Medium	Won
Lin Yun-Ju	LSA	Medium	Won
Morizono	LSA	Medium	Won
Achanta	RSA	Low	Won
Alamian	LSA	Low	Won
Lin Gaoyuan	LSA	High	Lost
Lebesson	LSA	Low	Won
Hirano	RSA	Medium	Won

**Figure 10:**
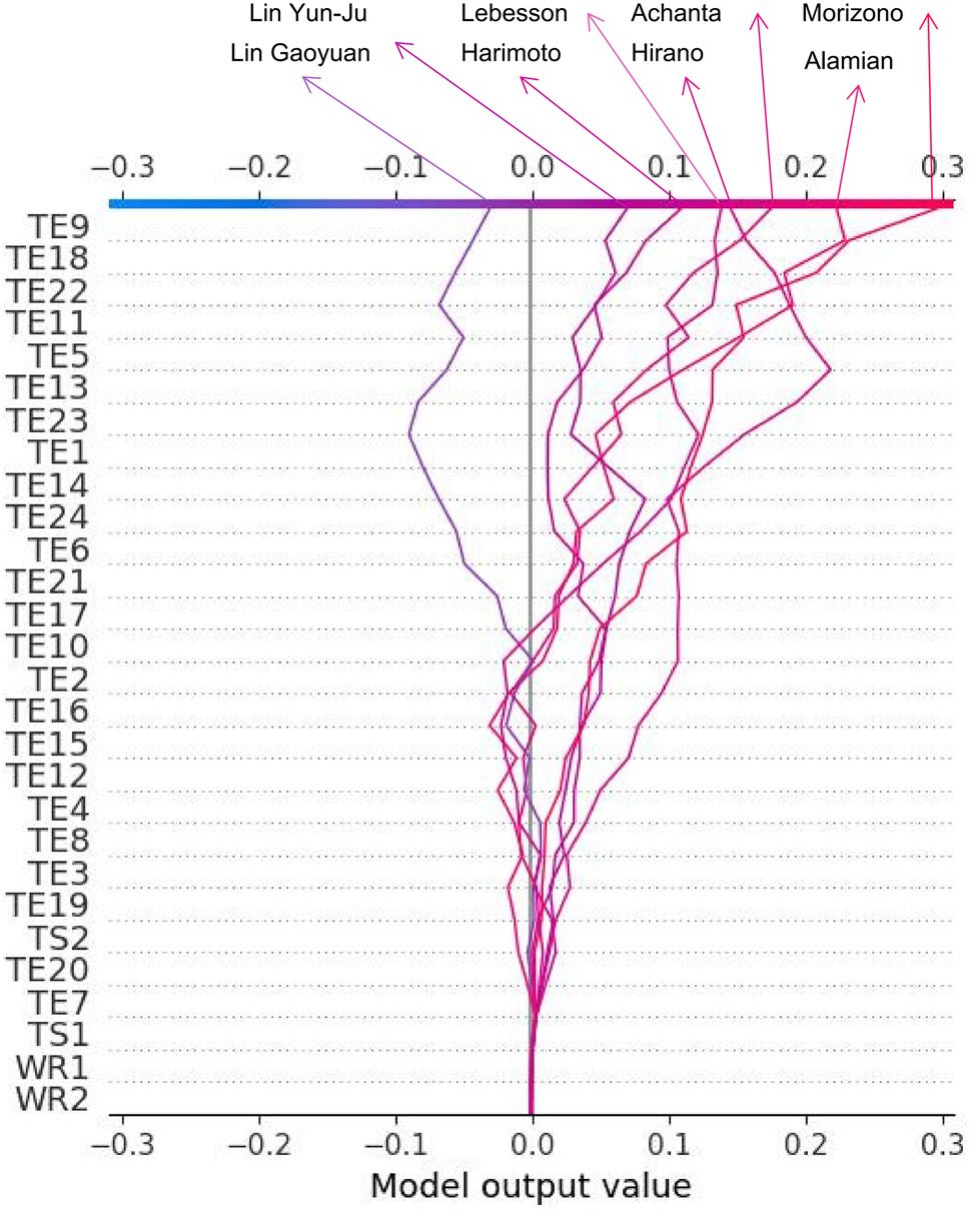
Using SHAP decision plot to diagnose Ovtcharov’s technical–tactical performance against his eight opponents. Figure is drawn by Python 3.8.8 (https://www.python.org/) and Microsoft Office 2019 (https://www.microsoft.com/). Abbreviation: TE technique effectiveness; TS1 player A’s technical style; TS2 player B’s technical Style; WR1 player A’s singles winning rate; TS2 player B’s singles winning rate.

As shown in Fig. 10, the SHAP method shows that TE9 ranks top, followed by TE18, TE22, TE11 and TE5, which illustrates the ESAP, ESI, and MSAP of Ovtcharov’s performance and the MRAP, ERAP, and ESAP of his opponents’ performance have a high impact on the MWP. According to the two players’ mutual correlations in Table 3, Fig. 10 implies that Ovtcharov’s performance in the ESAP-ERAP and MSAP-MRAP phases against his opponent greatly impacts the MWPs. Meanwhile, Fig. 10 shows that each feature’s contribution varies among different samples on the MWPs, which verifies that one player’s performance in matches is closely related to their opponents^10^ and that both sides in the match have interactive and logical relationships^10,28^.

**Figure 11:**
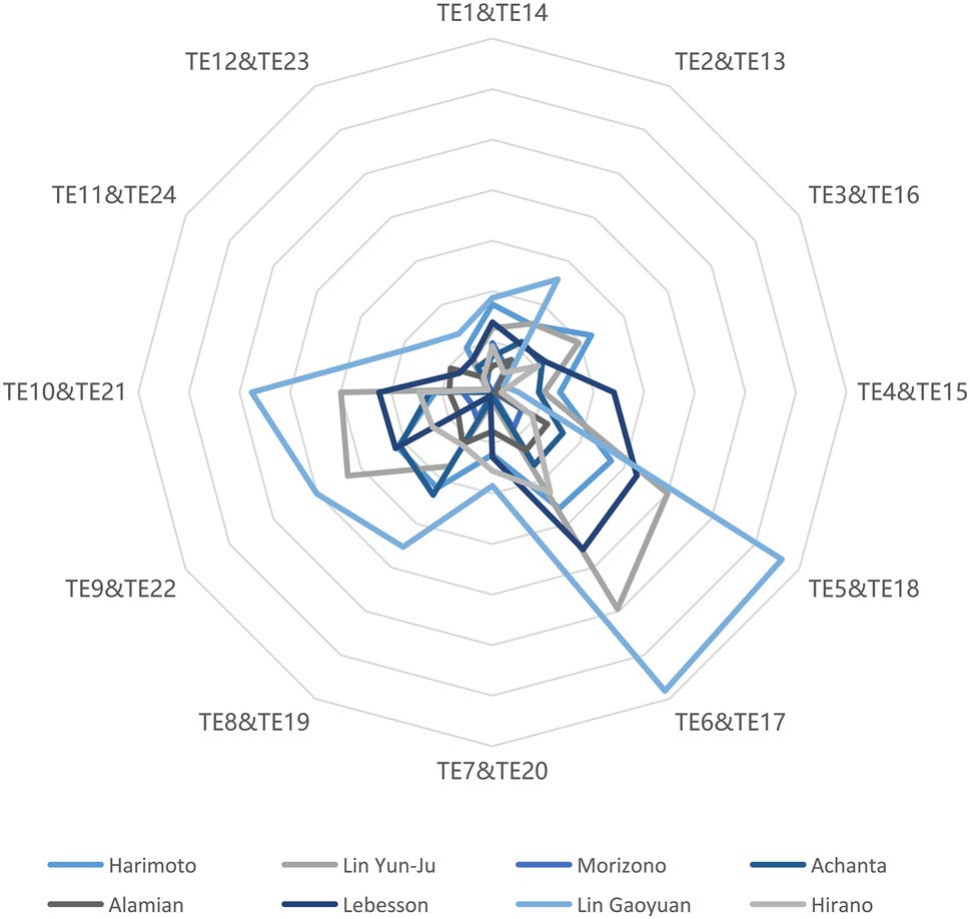
Using traditional simulation method to diagnose Ovtcharov’s technical–tactical performance against his eight opponents. Figure is drawn by Microsoft Office 2019 (https://www.microsoft.com/). Abbreviation: TE technique effectiveness.

As shown in Fig. 11, the traditional diagnosis method also shows that each weight differs among players on the MWPs. However, in Fig. 11, it is hard to know which phase is the most important among the eight matches, which can only be known as the most crucial phase in a single match rather than the whole match. Meanwhile, weight is calculated by the MWP’s value based on Eq. (5), and the weight’s size depends on the MWP’s size. Figure 11 shows that the results of the weight between Ovtcharov and Lin Gaoyuan are almost all higher than other diagnostic results, which illustrates that the importance of phases or features in different matches cannot be compared. Nevertheless, the SHAP method can fill this gap through local interpretation and analysis. As shown in Fig. 11, SHAP decision plots show how LSTM–BPNN arrives at each output. Therefore, the trend and the degree of impact on each feature’s respective output MWP in different matches can be identified based on the direction and slope of each curve.

**Figure 12:**
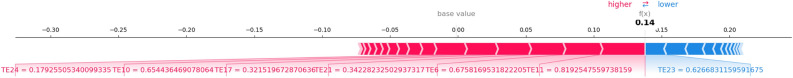
Using SHAP method to diagnose Ovtcharov’s technical–tactical performance against Harimoto. Figure is drawn by Python 3.8.8 (https://www.python.org/). Abbreviation: TE technique effectiveness.

To further investigate the difference between the traditional and the SHAP methods, this study conducts one match between Ovtcharov and Harimoto for analysis and diagnosis. Regarding the traditional diagnosis method, as shown in Table 6, the results show TE5&TE18 and TE6&TE17 have a high impact on the MWP and TE12&TE23, TE11&TE24 have a low impact on the MWP. As shown in Fig. 12, the results show TE11, TE6, TE21, TE17, TE10 and TE24 have a high and positive impact on the MWP, and TE23 has a high and negative impact on the MWP. In the diagnostic results of both methods, this study found that the traditional method cannot analyze the positive or negative impacts. Meanwhile, the two diagnosis methods have different analysis results, and the traditional one shows TE12&TE23 and TE11&TE24 have low impacts, but the SHAP method shows they all have high impacts on the MWP. To further analyze this phenomenon, the study finds that the traditional method presents this result because of Eq. (21). The weight values used in the traditional method to measure the importance of different features are obtained by simulating the input SRs by Eq. (21) and comparing the change in winning probabilities before and after the adjustment. According to Eq. (21), the value of the resulting increment y is lower when the input value SR converges to 1 or 0 compared to 0.5. Therefore, the simulated SR is also lower. Meanwhile, the simulated TE is also calculated from the simulated SR and the original UR. This indirect adjustment and simulation likely lead to the above results for adopting the traditional method. The black-box character of the models built by deep learning algorithms^60^ leads to traditional diagnosis methods relying heavily on such indirect simulation. However, giving direct feedback on the match conditions requires much work. The SHAP method used in this study is based on local interpretation, which enables the direct and objective reflection of the condition on the match without simulation.

**Table 6:** Using traditional simulation method to diagnose Ovtcharov’s technical–tactical performance against Harimoto.

	AMWP	Weight (%)	Ranking	Progression ranking
TE1&TE14	0.12508467	8.71	6	2
TE2&TE13	0.14760652	7.72	7	3
TE3&TE16	0.12156015	11.28	3	1
TE4&TE15	0.14616954	6.68	8	4
TE5&TE18	0.15558305	13.55	1	1
TE6&TE17	0.11888001	13.24	2	2
TE7&TE20	0.12857819	6.16	9	4
TE8&TE19	0.15197343	10.91	4	3
TE9&TE22	0.12217932	10.83	5	1
TE10&TE21	0.12925923	5.67	10	2
TE11&TE24	0.13618031	0.61	12	4
TE12&TE23	0.14400336	5.09	11	3

*TE* technique effectiveness.

Above all, the shortcoming of the traditional table tennis diagnostic method is that it cannot directly determine which feature has the most critical impact but only indirectly determines the feature importance on one match through the simulation analysis. Each feature’s importance on one or more matches, which can be obtained directly by the SHAP method, is more objective and reliable than the one revealed by the traditional simulation method.

### Limitation and Contribution

This study explores an innovative way to understand and analyze matches, and these results have implications for the performance analysis of table tennis and related racket sports. In detail, the special contributions of this work are: (1) This study proposed a hybrid technical–tactical analysis through the four-phase evaluation theory and the double three-phase evaluation theory and built a diagnosis model based on LSTM–BPNN; (2) This study used the SHAP to interpret the model’s output and diagnose the table tennis match; (3) The SHAP’s global interpretation of the model shows that the receive-attack and serve-attack phases of the ending match have an essential impact on the current tennis matches; (4) The local interpretation and case application found that the SHAP method and traditional simulation method came up with different diagnostic results. SHAP could directly obtain each feature’s importance on one or more matches, which is more objective and reliable than the traditional simulation method.

Although this study has made some contributions, there are some limitations. The data obtained from the hybrid analysis theory proposed in this study and previous analysis theories like the four-phase and double three-phase evaluation theory are all outcome-focus data. Although the technical–tactical diagnosis of this type of data can find the key phases that impacted the match based on artificial intelligence algorithms, it can’t reflect the progress and the antecedents and consequences of the critical phases. Therefore, further study is suggested to focus more on the progress analysis in matches and construct theoretical models for progress analysis. Moreover, many factors can affect the players’ technical and tactical performance in matches, including the players’ psychological and physiological factors, venue factors, equipment factors, etc. Further studies, which consider these factors, will need to be undertaken.

## Conclusion

The interpretation of the technical–tactical diagnosis model is essential for players and coaches to trust analysis results. This study uses the SHAP to interpret the model’s output and diagnose the table tennis match. Meanwhile, this study proposes a hybrid technical–tactical analysis theory through the four-phase and double three-phase evaluation theories and built an interactive diagnosis model based on a hybrid deep learning model, namely LSTM–BPNN. Our main findings demonstrates that the LSTM–BPNN achieves the best performance among the seven algorithms. Moreover, the global interpretation of the model shows that the receive-attack and serve-attack phases of the ending match have an essential impact on the current male tennis matches. The local interpretation and case application find that the SHAP and traditional simulation methods produce different diagnostic results. SHAP could directly obtain each feature’s importance on one or more matches, which is more objective and reliable than the traditional simulation method. These results have implications for the technical–tactical analysis theories, the diagnostic method, and algorithm selection for table tennis and related racket sports performance analysis.

## Supplementary Information

Supplementary Information.

## Data Availability

The datasets generated during the current study are not publicly available but are available from the corresponding author upon reasonable request.
