# Supplementary material for: Elite male table tennis matches diagnosis using SHAP and a hybrid LSTM–BPNN algorithm
Source: Sci Rep. 2023 Jul 17;13:11533. doi: 10.1038/s41598-023-37746-1 (PMC10352295; doi:10.1038/s41598-023-37746-1)
Supplement: Supplementary file 1 — Supplementary Information. [file 41598_2023_37746_MOESM1_ESM.pdf]

```python
class Lstm_BP(nn.Module):
    def __init__(self, input_size, hidden_size1, num_layers=1):
        super().__init__()
        self.lstm = nn.LSTM(input_size, hidden_size1, num_layers)
        self.hanshu1 = nn.ReLU()
        self.bpnn = nn.Linear(4 + 2 * 12 * hidden_size1, 40)
        self.hanshu2= nn.ReLU()
        self.line= nn.Linear(40,1)
    def forward(self,  train_x_tensor):
        s = train_x_tensor.shape
        player1 = train_x_tensor[:, :, 0:12].squeeze().transpose(0, 1).unsqueeze(-1)
        player2 = train_x_tensor[:, :, 12:24].squeeze().transpose(0, 1).unsqueeze(-1)
        mix =  train_x_tensor[:, :, 24:].squeeze()
        output1, _ = self.lstm(player1)
        output3_ = output1.transpose(0, 1).contiguous().view(s[0], -1)
        output2, _ = self.lstm(player2)
        output4_ = output2.transpose(0, 1).contiguous().view(s[0], -1)
        output3= self.hanshu1(output3_)
        output4 =self.hanshu1(output4_)
        bpinput = torch.cat([mix, output3, output4], dim=-1)
        output5 = self.bpnn(bpinput)
        output6 = self.hanshu2(output5)
        output =  self.line(output6)
        return output
```
